# Silver nanoparticles disrupt germline stem cell maintenance in the *Drosophila* testis

**DOI:** 10.1038/srep20632

**Published:** 2016-02-05

**Authors:** Cynthia Ong, Qian Ying Lee, Yu Cai, Xiaoli Liu, Jun Ding, Lin-Yue Lanry Yung, Boon-Huat Bay, Gyeong-Hun Baeg

**Affiliations:** 1 Department of Anatomy, Yong Loo Lin School of Medicine, National University of Singapore, Singapore 117594, Singapore; 2 Temasek Life Sciences Laboratory, National University of Singapore, Singapore 117604, Singapore; 3 Department of Biological Sciences, National University of Singapore, Singapore 117543, Singapore; 4 Department of Materials Science & Engineering, Faculty of Engineering, National University of Singapore, 7 Engineering Drive 1, 117574, Singapore; 5 Department of Chemical & Biomolecular Engineering, Faculty of Engineering, National University of Singapore, Singapore 117576, Singapore

## Abstract

Silver nanoparticles (AgNPs), one of the most popular nanomaterials, are commonly used in consumer products and biomedical devices, despite their potential toxicity. Recently, AgNP exposure was reported to be associated with male reproductive toxicity in mammalian models. However, there is still a limited understanding of the effects of AgNPs on spermatogenesis. The fruit fly *Drosophila* testis is an excellent *in vivo* model to elucidate the mechanisms underlying AgNP-induced defects in spermatogenesis, as germ lineages can be easily identified and imaged. In this study, we evaluated AgNP-mediated toxicity on spermatogenesis by feeding *Drosophila* with AgNPs at various concentrations. We first observed a dose-dependent uptake of AgNPs *in vivo*. Concomitantly, AgNP exposure caused a significant decrease in the viability and delay in the development of *Drosophila* in a dose-dependent manner. Furthermore, AgNP-treated male flies showed a reduction in fecundity, and the resulting testes contained a decreased number of germline stem cells (GSCs) compared to controls. Interestingly, testes exposed to AgNPs exhibited a dramatic increase in reactive oxygen species levels and showed precocious GSC differentiation. Taken together, our study suggests that AgNP exposure may increase ROS levels in the *Drosophila* testis, leading to a reduction of GSC number by promoting premature GSC differentiation.

The incorporation of nanomaterials in a wide variety of biomedical and daily products is becoming increasingly common today. The minute size of nanomaterials (1–100 nanometers) with a high surface area to volume ratio, contribute to distinctive properties that are different when nanomaterials are in their bulk form^1^,^2^. According to the Woodrow Wilson database of nanotechnology-based products, the most popular nanomaterial today is silver nanoparticles (AgNPs)^3^. AgNPs have been developed as a cancer diagnostic tool due to their strong absorbance and scattering properties. AgNPs have been shown to be more capable of accumulating at tumor sites than normal tissues due to the presence of leaky blood vessels and dysfunctional lymphatic vessels of tumors (also known as enhanced permeability and retention effect). Therefore, AgNPs can potentially be administered intravenously in cancer patients to provide excellent photoacoustic contrast with normal tissues, allowing a more accurate detection of tumors *in vivo*^4^. The antimicrobial property of AgNPs also makes them attractive for various medical applications. The coating of AgNPs on surgical instruments was found to induce bactericidal effects, thereby, inhibiting the formation of bacterial biofilm and reducing the number of hospital-acquired infections^5^,^6^. AgNPs can also be infused into synthetic tissue adhesives for ophthalmic applications, resulting in antibacterial properties and greater mechanical strength^7^.

However, studies have revealed that AgNPs could induce reactive oxygen species (ROS) production, followed by DNA fragmentation and mitochondrial damage, in a variety of cultured cell lines and organs such as liver, lungs and kidneys where AgNPs normally accumulate^8^,^9^,^10^. Recent studies have also revealed that AgNP exposure can cause male reproductive toxicity in mammals. For example, AgNPs were found to affect the proliferation of mouse spermatogonial stem cells by disrupting GDNF/Fyn kinase signaling^11^. The number of spermatogenic cells was also adversely affected by AgNPs in a rat model^12^. Daily oral exposure of prepubertal Wistar rats to AgNPs resulted in sperm abnormalities, such as the reduction in mitochondrial activity and damage inflicted on the sperm plasma membrane and acrosome^13^. In addition, single intravenous injections of AgNPs in mice can also induce a significant effect on germ cells, adversely affecting spermatocytes development^14^. Despite studies being conducted on the effects of AgNPs on male fertility, the exact molecular mechanisms underlying the reduced male fertility by AgNP exposure are not fully understood. The adult male *Drosophila melanogaster* contains a pair of testes. Each is a tubular structure with germline stem cells (GSCs) and somatic cyst stem cells (CySCs) residing in a stem cell niche, which is composed of a small group of stromal cells^15^. The *Drosophila* testis is a useful *in vivo* model for studying the behavior of GSCs, as they are easily identified, traced, imaged and genetically manipulated compared to those in complicated mammalian models. Thus, *Drosophila*, with its low maintenance cost, a short life cycle, distinct developmental stages and physiological similarity to humans, can be used as an excellent model organism to investigate AgNP-induced male reproductive toxicity^16^,^17^.

In this study, we demonstrated that AgNPs are taken up through ingestion and accumulated in a dose-dependent manner in *Drosophila*. Toxicity of AgNPs was evidenced by a decrease in the viability and a delay in the development of *Drosophila*. Interestingly, a significant decline in male fecundity was found after AgNP exposure. Further investigations revealed that AgNPs induce excessive amounts of ROS in the niche-GSCs, resulting in a decrease in GSC number mediated by promoting precocious GSC differentiation. Our findings have provided biological insights into the toxicity of AgNPs in spermatogenesis, highlighting the importance of risk assessment of AgNPs when used in consumer or biomedical applications.

## Results and Discussion

The fruit fly *Drosophila* has a short life cycle, distinct development stages, physiological similarity to humans, and a streamlined genome with high levels of gene conservation. Furthermore, numerous genetic tools and reagents for the study of complex biological processes are available in *Drosophila*^18^,^19^. Consistent with the advantages of using *Drosophila* as a reliable *in vivo* model organism, there is a growing body of research which attempts to understand nanomaterial-mediated toxicity using *Drosophila*. In this study, we attempted to elucidate the mechanisms underlying AgNP-mediated toxicity on *Drosophila* spermatogenesis.

### Characterization of AgNPs

**Figure 1:**
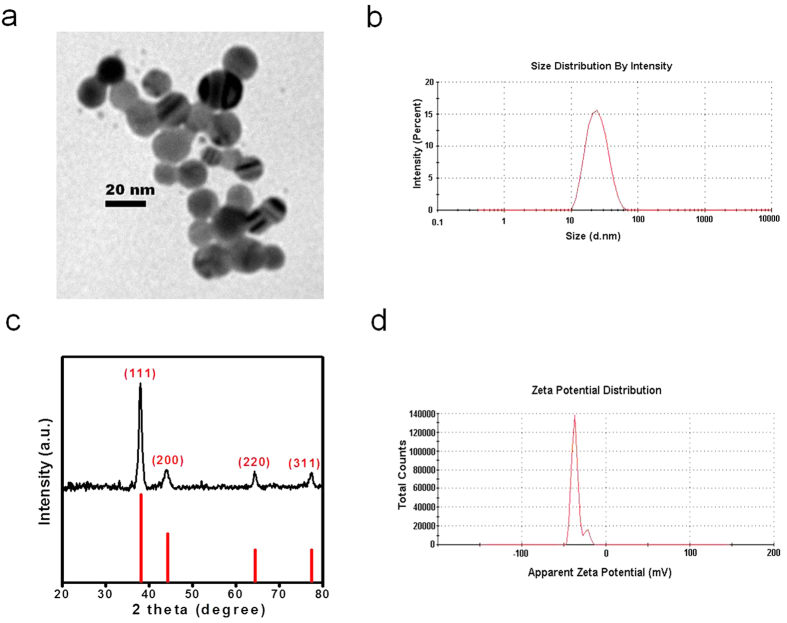
Characterization of AgNPs. (**a**) TEM micrographs show spherical AgNPs. (**b**) DLS measurement of AgNPs reveals a mean diameter of ~20 nm. (**c**) All diffraction peaks can be exclusively indexed as face-centred cubic (fcc) Ag (JCPDS PDF No.01-087-0717), corresponding to (111), (200), (220) and (311). By applying the Scherrer equation to the most intense peak of the XRD pattern, the mean crystallite size is estimated to be about 11 nm. (**d**) The zeta potential of AgNPs is −35.7 mV.

Transmission electron microscopy (TEM) shows that the spherical shaped NPs were fairly uniform in size (Fig. 1a). Dynamic light scattering (DLS), which measures the hydrodynamic diameter of AgNPs in suspension, revealed that the AgNPs were monodispersed in aqueous solution and ~20 nm in size as indicated by the single peak (Fig. 1b). The crystal structure of the AgNPs was identified by using X-ray powder diffraction (XRD). All diffraction peaks could be exclusively indexed as face-centred cubic (fcc) Ag (JCPDS PDF No.01-087-0717), indicating high crystallinity. By applying the Scherrer equation to the most intense peak of the XRD pattern, the mean crystallite size was estimated to be about 11 nm (Fig. 1c). The zeta potential of the AgNPs was −35.7 mV, implying that the AgNPs were moderately stable (Fig. 1d).

### AgNPs induce toxic effects in the fruit fly *Drosophila*

**Figure 2:**
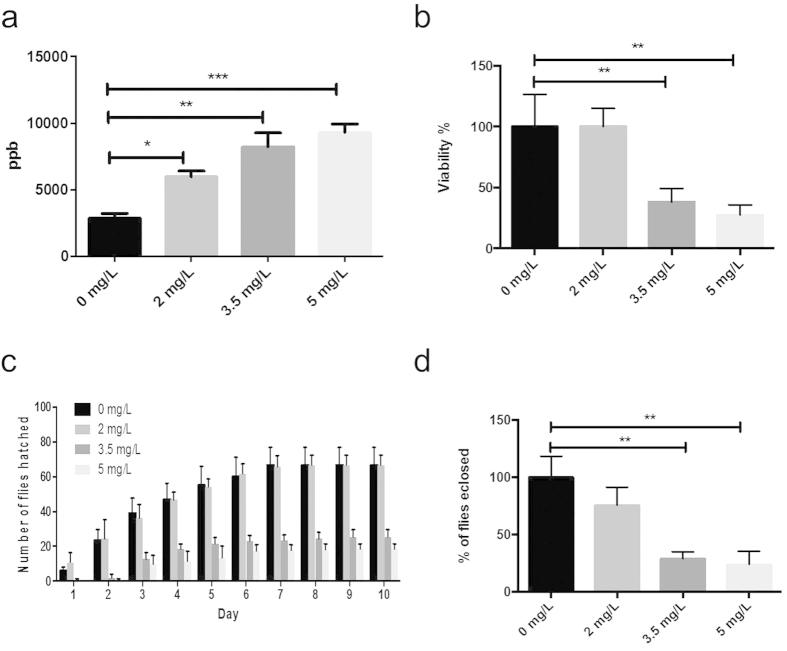
AgNPs induce toxic effects in *Drosophila*. (**a**) AgNP-fed *Drosophila* shows a dose-dependent uptake and accumulation of AgNPs. (**b**) A dose-dependent decline in the viability of *Drosophila* upon AgNP ingestion. (**c**) A delay in development is observed in AgNP-fed *Drosophila*. *Drosophila* treated with higher dose of AgNPs (3.5 mg/L or 5 mg/L) has the highest eclosion percentage of F1 offsprings on day 3, while most of control F1 offsprings (0 mg/L) eclose on day 2. (**d**) A decline in the fecundity of F1 males exposed to AgNPs is observed in a dose-dependent manner. Error bar = SEM, *p-value < 0.05; **p-value< 0.01; ***p-value < 0.001.

To examine the potential toxic effects of AgNPs in *Drosophila*, we first monitored the accumulation of AgNPs *in vivo* after exposure. Flies were treated in food containing AgNPs at various concentrations, ranging from 0 mg/L to 5 mg/L. Inductively coupled plasma mass spectrometry (ICP-MS) analysis showed that AgNPs accumulated in a dose-dependent manner *in vivo* (Fig. 2a). Ag accumulation after ingestion of AgNPs has also been reported in other studies using atomic absorption analysis^20^,^21^. Correspondingly, a dose-dependent decrease in the viability of *Drosophila* was observed (Fig. 2b). A significantly less number of F1 (1^st^ filial or generation) flies was able to successfully eclose upon exposure to higher doses of AgNPs (3.5 mg/L or 5 mg/L) as compared to control flies. Treatment of AgNPs also resulted in a delay in the developmental process of *Drosophila*. As shown in Fig. 2c, flies exposed to AgNPs at higher concentrations such as 3.5 mg/L or 5 mg/L showed a delayed eclosion compared to those exposed to 0 mg/L or 2 mg/L AgNPs. These findings are in accord with previous reports that AgNPs are indeed ingested by *Drosophila* with subsequent toxic effects^22^,^23^,^24^,^25^,^26^,^27^. Furthermore, we found that the fecundity of F1 males was adversely affected by the ingestion of AgNPs (Fig. 2d). A gradual decline in male fecundity was observed with increasing AgNP concentration. The number of F2 flies derived from F1 males, which were previously exposed to 3.5 mg/L or 5 mg/L of AgNPs, was significantly reduced compared to that from control F1 males. Specific effects of AgNPs on reproduction have previously been reported in mammals, as well as in *Drosophila*. In mammals, the epididymal sperm count was found to be significantly decreased in rats after intravenous administration of AgNPs. DNA damage was also observed in germ cells after AgNP injection^28^. Furthermore, even during prepubertal oral exposure of AgNPs to rats, reproductive parameters such as sperm morphology, acrosome, plasma membrane integrity and mitochondrial activity were found to be adversely affected in adulthood^13^. On the other hand, fertility toxicity studies in *Drosophila* have shown that the exposure of AgNPs to both male and female flies reduces mating success, leading to a decrease in the number of F2 and F3 offsprings, with an improvement in fecundity from F4 onwards^21^,^23^,^24^. Nonetheless, the limited understanding of the mechanisms of the reproductive toxicity induced by AgNPs would need further elucidation in order to have meaningful assessment of nanosafety.

### AgNPs decrease germline stem cell number in the *Drosophila* testes

**Figure 3:**
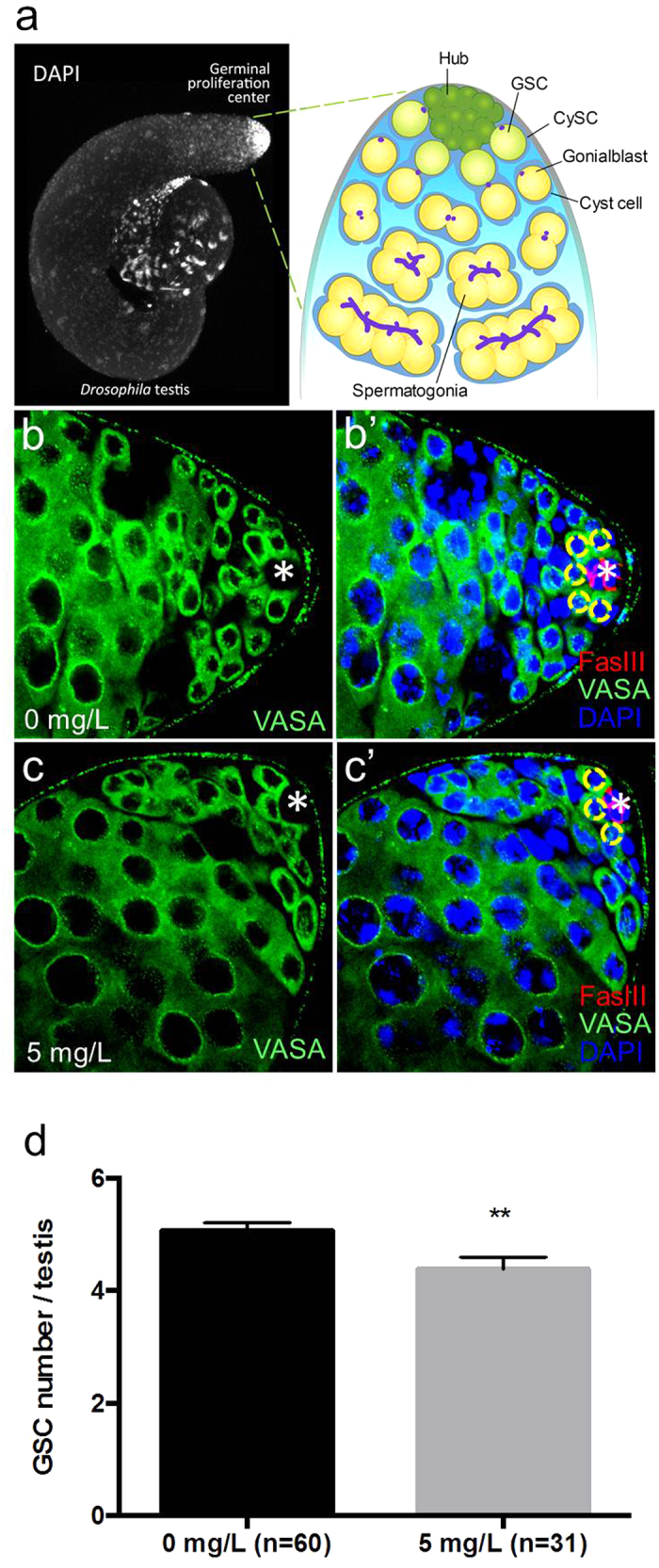
AgNPs decrease germline stem cell number in the Drosophila testis. (**a**) Testis stained with DAPI, and testis schematic. Immunofluorescence staining shows GSCs attached to the somatic hub cells in testis exposed to 0 mg/L AgNPs (**b,b’**) and in testis exposed to 5 mg/L AgNPs (**c,c’**). Hub cells are marked with * and GSCs are delineated by yellow circles. (**d**) Testes treated with 5 mg/L AgNPs show a decreased number of GSCs compared to controls. Error bar = SEM, **p-value < 0.01.

DAPI (4′,6-diamidino-2-phenylindole) staining marks mitotically-active cells at the apical tip of the testis, where germline stem cells (GSCs) and somatic cyst stem cells (CySCs) reside in a stem cell niche (microenvironment), which is composed of a small group of post-mitotic cells, known as hub cells (Fig. 3a). GSCs in the adult male *Drosophila* divide asymmetrically to produce one daughter cell with stem cell identity and the other, gonialblast, which further differentiates and eventually gives rise to sperms. On the other hand, CySCs are somatic stem cells that contribute to the GSC niche. Two CySCs are known to envelop each GSC and produce as-yet-unknown signal(s) to determine the self-renewal and differentiation of the GSC. Their daughter cells, called cyst cells, are known to accompany and co-differentiate with the germ cells that they enveloped throughout the entire spermatogenesis^29^,^30^ and thus functionally resemble Sertoli cells in the mammalian testes^31^. Indeed, there are several similarities between mice and *Drosophila* GSCs. For instance, the behavior of mammalian GSCs is influenced by signal(s) produced from the neighboring somatic Sertoli cells, and transit amplifying germ cells remain interconnected and divide in synchrony. However, unlike mammalian GSCs, those of *Drosophila* can be easily imaged, traced and genetically manipulated, thus making the *Drosophila* an increasingly popular model organism for studying the reproductive toxic effects of nanomaterials. In this study, we have shown a dose-dependent decline in the fecundity of F1 male flies exposed to AgNPs at various concentrations. Further investigations revealed that poor male fecundity is related to a decrease in the number of GSCs attached to hub cells in treated testis. Typically, 5–9 GSCs surround the hub cells in each testis^32^. The testes of F1 male flies exposed to either 0 mg/L (n = 60) or 5 mg/L (n = 31) AgNPs were dissected out, and subsequently immunostained with an anti-Fasciclin III antibody (marks hub cells) and an anti-Vasa antibody (marks GSCs and differentiating germ lineages). In the testes of flies exposed to 0 mg/L AgNPs (n = 60), 5.07 GSCs were observed. However, only 4.39 GSCs were counted in testes of flies exposed to 5 mg/L AgNPs (n = 31). We found a significant decrease in the number of GSCs attached to hub cells in testes of F1 male flies treated with 5 mg/L AgNPs compared to controls (Fig. 3b–d). Since GSCs give rise to sperms, the decrease in GSC number could possibly have adversely affected sperm production and lead to a decline in male fertility. It would appear that exposure to AgNPs could result in a decrease in GSC number in the *Drosophila* testis.

### AgNPs induce oxidative stress in the *Drosophila* testis

**Figure 4:**
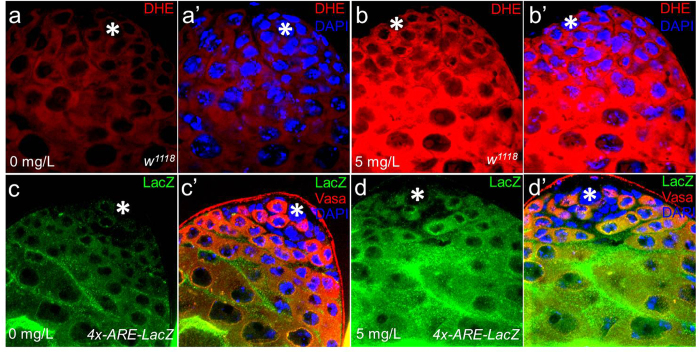
AgNPs induce oxidative stress in the *Drosophila* testis. (**a,a’**) DHE staining of control testis shows the basal level of ROS at the apical tip, in which GSCs and early germ cells reside, suggesting that GSCs maintain low ROS. (**b,b’**) In testes treated with 5 mg/L AgNPs, a dramatic increase in the fluorescent intensity of DHE staining is observed that is indicative of excessive ROS production. Transgenic flies harboring the oxidative stress reporter gene *4xARE-LacZ* were also used to examine the effects of AgNPs on ROS production. While control testis shows weak LacZ staining at the apical tip (**c,c’**), AgNP-treated testis shows enhanced LacZ staining (**d,d’**). Altogether, these observations strongly indicate that AgNPs induce ROS in the *Drosophila* testis. Hub cells are marked with *.

To understand the mechanisms underlying the reduction of GSC number by AgNPs, we next examined the effects of AgNPs on reactive oxygen species (ROS) production in the *Drosophila* testis. ROS, which are commonly induced by AgNPs, have been implicated as the primary cause of AgNP-mediated toxicity^33^,^34^,^35^. Since low levels of ROS are essential for stem cells to keep their stem cell identity, it is conceivable that aberrant ROS levels affected GSC behavior in AgNP-treated testes. In support of this, previous studies have suggested that high ROS levels are closely associated with infertility by causing damaging effects on spermatogenesis in humans^36^,^37^. Dihydroethidium (DHE) was used to monitor ROS levels as it readily reacts with superoxide anions to form 2-hydroxyethidium, generating red fluorescence^38^. In control testes of flies treated with 0 mg/L AgNPs, low amounts of ROS were detected at the apical tip of the testis. On the other hand, intermediate levels of ROS were observed in cells that are considered to be differentiating germ cells, such as spermatogonia and spermatocytes (based on their size and morphology), which are located several cell diameters away from hub cells (Fig. 4a,a’). However, the testes of 5 mg/L AgNP-treated *Drosophila* showed a dramatic increase in the levels of ROS at the apical tip of the testis, where GSCs and early germ cells (such as gonialblasts) are present (Fig. 4b,b’). To verify the effects of AgNPs on ROS production, we next used transgenic flies carrying the independent oxidative stress reporter gene *4xARE* (antioxidant response element)-*LacZ*^39^, and assessed whether AgNP exposure could induce the reporter activity. During oxidative stress, the Maf/Nrf2 dimer is known to bind to ARE, leading to a transcriptional up-regulation of antioxidant genes^40^,^41^. Henceforth upon greater oxidative stress, high LacZ expression is expected. In support of the finding from DHE staining, a low level of LacZ expression was detected at the apical tip of control testis (Fig. 4c,c’). However, we observed high LacZ expression at the apical tip of treated testis (Fig. 4d,d’). These observations strongly suggest that AgNP exposure can induce ROS production associated with a decrease in GSCs in the *Drosophila* testis.

### AgNP exposure causes precocious differentiation of germline stem cells

**Figure 5:**
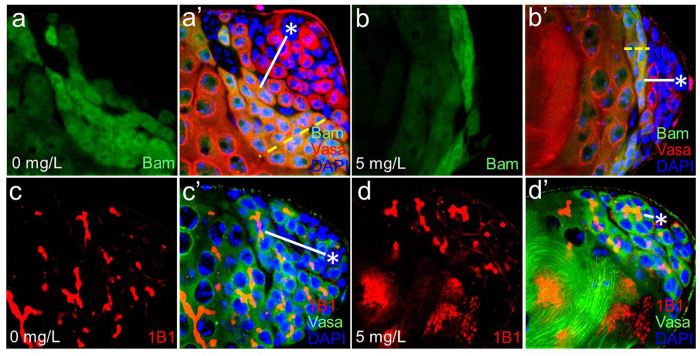
AgNPs cause precocious differentiation of germline stem cells in the *Drosophila* testes. (**a,a’**) Spermatogonia positive to the differentiation factor Bam are located several cell diameters away from hub cells in control testis (white line indicates the distance between hub cells and Bam-positive spermatogonia). (**b,b’**) In treated testis, Bam-positive spermatogonia are detected close to hub cells, an indication of premature GSC differentiation (compare the length of white lines in (**a’,b’**). Notably, more layers of Bam-positive cells are observed in control testis compared to those in treated testis, suggesting that AgNPs decrease transit amplifying spermatogonia (yellow dotted lines delineate the layers of spermatogonia). (**c,c’**) In control testis, branching fusomes are detected in differentiating spermatogonia located several cell diameters away from hub cells (white line highlights the distance between hub cells and spermatogonia with branching fusome). (**d,d’**) In treated testis, branching fusomes are often detected next to hub cells (compare the length of white lines in (**c’,d’**). Hub cells are marked with *.

Normal stem cells are known to reside in stem cell niches, which are characterized by a low-moderate ROS environment so that stem cells remain in a quiescent state, a property that is essential for the self-renewal capacity of stem cells^42^,^43^. Oxidative stress has been reported to influence the self-renewal and differentiation of stem cells in various model organisms. Human hematopoietic stem cells lose their self-renewal capacity and instead undergo differentiation in a high ROS environment^44^,^45^. Similarly, in neural stem cells, lower ROS levels were found to have enhanced self-renewal capacities of the cells^46^. Furthermore, in the *Drosophila* hematopoietic stem cell niche, high ROS were shown to prime the hematopoietic progenitors for differentiation, where an increase in ROS exceeding the basal level promotes the differentiation of hematopoietic precursor cells into mature blood cell types such as plasmatocytes, crystal cells and lamellocytes^47^. These studies suggest that the excessive amounts of ROS can accelerate the differentiation of stem cells. Thus, we hypothesized that the reduction of GSCs results from precocious GSC differentiation, mediated by AgNP-induced high ROS. To test this hypothesis, we examined the expression pattern of the differentiation factor Bag-of-marbles (Bam; marks transit amplifying spermatogonia), which functions to stop the spermatogonial transit amplifying divisions and promote the spermatocyte differentiation of germ cells^48^,^49^,^50^. In control testes, Bam was normally detected in 4, 8, and early 16-germ cell located several cell diameters away from hub cells (Fig. 5a,a’; white line highlights the distance between hub cells and Bam-positive spermatogonia). However, in the testes of 5 mg/L AgNP-treated *Drosophila*, Bam-positive spermatogonia were often detected next to hub cells (Fig. 5b,b’), and much less Bam-positive spermatogonia were found compared with control (compare Fig. 5a,b). This strongly suggests that GSCs with high ROS have lost their ability to self-renew and instead underwent the process of premature differentiation. For further confirmation, we examined the morphology of the fusome, which is an organelle specific to germ cells that appears spheroid throughout GSCs and GSC-gonialblast pairs, but branches throughout spermatogonia due to the incomplete cytokinesis^51^. In control testes, spherical fusomes, also known as spectrosomes, were observed in GSCs attached to hub cells and in GSC-gonialblast pairs, whereas branching fusomes were detected in differentiating spermatogonia located several cell diameters away from hub cells (Fig. 5c,c’). However, in testes of flies treated with 5 mg/L AgNPs, less spherical fusomes compared with control were observed, suggesting that AgNPs reduced GSC number. Importantly, branching fusomes were detected close to hub cells (Fig. 5d,d’). These findings indicate that AgNP treatment causes a decrease in GSCs by promoting premature differentiation of GSCs. This precocious GSC differentiation may disrupt GSC maintenance, decreasing the GSC pool and eventually lowering the number of sperms produced.

### AgNPs decrease the proliferation of spermatogonia in the *Drosophila* testes

**Figure 6:**
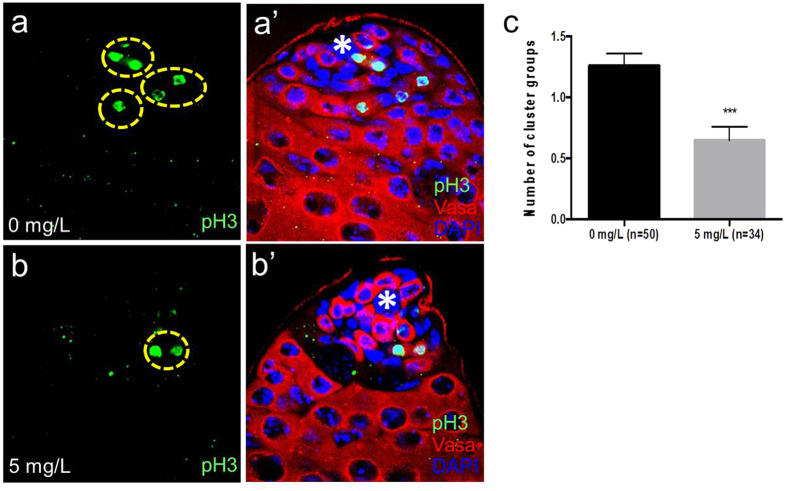
AgNPs decrease the number of transit amplifying spermatogonia in the *Drosophila* testes. Immunostaining for phospho-histone H3 was performed to mark transit amplifying spermatogonia in control testis (**a,a’**) and treated testis (**b,b’**). Compared to the number of spermatogonial clusters (cysts) positive to phospho-histone H3 in control testis, a much lower number of cysts is observed in treated testis (**c**). Error bar = SEM, ***p-value < 0.001.

Gonialblast undergoes four rounds of transit amplifying mitotic divisions to produce 16 spermatogonia. Due to the incomplete cytokinesis, the 16 spermatogonia are connected by intercellular bridges, called ring canals. The 16 cells then differentiate into spermatocytes, and undergo terminal differentiation via meiosis to form spermatids and subsequently mature sperms^15^,^32^,^52^. Therefore, it is conceivable that a reduction of transit amplifying cell divisions may lead to a decrease in the number of sperms produced, thereby negatively affecting male fertility. Since the testis of flies treated with 5 mg/L AgNPs contained much thinner cell layers of Bam-positive spermatogonia compared with control (compare yellow lines in Fig. 5a’,b’), we reasoned that AgNPs also disrupt the transit amplifying mitotic divisions of gonialblasts by promoting the premature differentiation of spermatogonia into spermatocytes. After exposure of flies to 5 mg/L AgNPs, the testes were found to have a lesser number of Bam-positive TA cells compared to controls (Fig. 5a,b), signifying that AgNPs can cause a reduction of TA cells. In addition, we performed immunostaining on the testes of treated flies with an antibody against phospho-histone H3, which can serve as a marker specific for mitotic cells. Consistently, we found that testes of flies exposed to AgNPs had significantly less clusters of mitotically-active phospho-histone H3-positive TA cells compared to controls (Fig. 6). This suggests that AgNPs also result in a premature termination of TA divisions, leading to less sperm production that could attribute to a decrease in male fertility. The epidermal growth factor receptor (EGFR) cascade is one of the intrinsic signaling pathways that governs germ cell differentiation in the *Drosophila* testis. Activation of EGFR signaling in testis has been shown to restrict GSC self-renewal and instead promote differentiation^53^. In addition, it was reported that in *raf*-deficient testes, spermatogonia do not transit to the spermatocyte stage due to the blockage of spermatogonia differentiation^54^. These suggest that high ROS induced by AgNPs may activate EGFR signaling and thus promote the premature differentiation of germ lineages. Notably, AgNPs are known to result in lower motility, curvilinear velocity and oxygen consumption of sperms in rabbits exposed to AgNPs through intravenous injection. Hence, we cannot exclude the possibility that AgNPs could also affect the functional integrity of sperms^55^.

## Conclusions

Administration of AgNPs has resulted in a significant toxicity in *Drosophila*. After ingestion of AgNPs, a dose-dependent accumulation of Ag was observed in the flies. Increased exposure to AgNPs caused a decline in survival and a delay in the development of the F1 offsprings. The fecundity of F1 male flies was also adversely affected by AgNPs. Importantly, we found that AgNPs induce ROS production in the *Drosophila* testis, which subsequently lead to a decrease in GSCs and TA cells via enhancement of the premature differentiation of the cells. Hence, reduced GSC and TA cell numbers could attribute at least in part to a decrease in the fecundity of AgNP-exposed F1 males.

## Methods

### Synthesis and characterization of AgNPs

AgNPs of size 20 nm were synthesized by the reduction of silver nitrate. Briefly, aqueous silver nitrate, tribasic sodium citrate dehydrate and boiling ionized water were added while stirring followed by the dropwise addition of aqueous sodium borohydride. Solutions containing AgNPs, were cooled and then purified by ultrafiltration with a 10,000 Da molecular weight cut-off regenerated cellulose filters. Unreacted reagents were removed as filtrate while AgNPs were retained as retentate. TEM of AgNPs was carried out by dropping colloidal AgNPs on copper grids pre-coated with Formvar and viewed with the JEOL JEM 1010 Transmission Electron Microscope. DLS (Zetasizer Nano ZS, UK) was used to investigate the hydrodynamic size of AgNPs in solution and the zeta potential was also determined. The Bruker D8 Advanced Diffractometer System equipped with Cu/Kα radiation in the 2θ range from 20° to 80° (λ = 1.5418 Å) was used to study the XRD pattern of AgNPs.

### Fly Strains

Fly stocks were maintained under standard culture conditions. *w*^*1118*^ (a white eyed stock) flies were obtained from the Bloomington *Drosophila* Stock Center and used for viability, development and fecundity assays. Transgenic flies carrying a *4xARE-LacZ* reporter gene were used to examine the effects of AgNPs on ROS production (a gift from Dr. Dirk Bohmann; Sykiotis and Bohmann, 2008^39^). Transgenic flies carrying a *GFP* reporter gene, which is transcriptionally regulated by the promoter of *bam* (*bag of marbles*), were used to monitor the differentiation of GSCs (a gift from Dr. Dennis McKearin; Chen and McKearin, 2003^56^).

### AgNPs exposure to *Drosophila*

AgNPs at a concentration of 2 mg/L, 3.5 mg/L or 5 mg/L were added to *Drosophila* food, which contains cornmeal flour, dextrose, brewer’s yeast, Bacta agar and Nipagin. Unreacted reagents that were removed as filtrate during synthesis were added to *Drosophila* food as a control (0 mg/L). Both male and female flies were added to vials with either AgNP-food or control food. The parent flies were then removed after five days. Newly laid eggs were treated with AgNPs until they eclosed (embryonic stage to adult stage).

### Inductively coupled plasma-mass spectrometry (ICP-MS) of *Drosophila*

Ten whole male flies were homogenized in water using a homogenizer and then acid- digested for 24 hours by Aqua Regia. The samples were then diluted with deionized water to appropriate volumes for triplicate analyses using the Agilent 7500 Series ICP-MS (Perkin Elmer, USA). Three independent experiments were carried out.

### Viability, development and fecundity of *Drosophila* after AgNP treatment

The effects of AgNPs on *Drosophila* viability were evaluated by counting the number of successfully eclosed F1 flies. The number of days required for the first eclosion was recorded to study the effects of AgNPs on developmental process. To examine the fecundity of F1 males, newly eclosed F1 males exposed to AgNPs were collected and added to normal food for 24 hours for recovery. 6 virgin female flies (unexposed to AgNPs) were then allowed to mate with the F1 male for 24 hours. The male flies were then removed after 24 hours, and only female flies were left to lay eggs. The number of F2 flies eclosed was then counted. Three independent experiments were carried out for statistical analysis.

### Whole-mount immunofluorescence of the *Drosophila* testis

The *Drosophila* testes were dissected in dissecting solution pH 7.2 (130 mM NaCl; 1.9 mM CaCl_2_; 4.7 mM KCl; 10 mM HEPES), fixed in 4% paraformaldehyde for 20 minutes and washed three times for 20 minutes each with PBST (PBS with 0.1% Triton-X). The resulting testes were then incubated with primary antibodies overnight at 4 °C. The primary antibodies used were; mouse anti-Fasciclin III monoclonal antibody (1:1000, Developmental Studies Hybridoma Bank; DSHB), rat anti-Vasa monoclonal antibody (1:100, DSHB), mouse anti-LacZ monoclonal antibody (1:2,000, Sigma), mouse anti-Bam monoclonal antibody (1:100, DSHB), and rabbit anti-phospho-histone H3 antibody (1:200, Cell Signaling). The testes were washed three times for 20 minutes each with PBST and then incubated with secondary antibodies for 2 hours at room temperature. The secondary antibodies used were; Cy3-conjugated rabbit anti-mouse antibody (1:300), Cy3-conjugated goat anti-rat antibody (1:300), FITC-conjugated rabbit anti-mouse antibody (1:300), and FITC-conjugated goat anti-rabbit antibody (1:300, Jackson laboratories). To detect reactive oxygen species, 30 μM dihydroethidium (DHE) was used to stain the dissected testis after fixation for 5 minutes and washed in PBST three times. The testes were then fine dissected, mounted on Vectashield mounting medium containing DAPI on a slide glass, and viewed under the confocal microscope (Olympus Fluoview FV1000 cLSM, Japan).

### Counting of germline stem cell number

The effect of AgNPs on the maintenance of GSCs in the *Drosophila* testis was studied. Testes from AgNP-treated F1 males were dissected out, and immunostained with DAPI (marks nuclei of cells), an anti-Fasciclin III antibody (marks hub cells) and an anti-Vasa antibody (marks germ lineages). The number of GSCs, which are attached to hub cells, was counted.

### Statistical Analysis

Statistical analyses were performed using the GraphPad Prism 6.0 software. Values from all experiments were expressed in mean ± standard error of the mean. The data was analyzed by unpaired t-test or one-way ANOVA with post-hoc test (Tukey’s Multiple Comparison Test). *p *< 0.05 was considered to be statistically significant.

## Additional Information

**How to cite this article**: Ong, C. *et al.* Silver nanoparticles disrupt germline stem cell maintenance in the *Drosophila* testis. *Sci. Rep.* **6**, 20632; doi: 10.1038/srep20632 (2016).
